# Coupling Resistive Switching Devices with Neurons: State of the Art and Perspectives

**DOI:** 10.3389/fnins.2017.00070

**Published:** 2017-02-15

**Authors:** Alessandro Chiolerio, Michela Chiappalone, Paolo Ariano, Sergio Bocchini

**Affiliations:** ^1^Center for Sustainable Future Technologies, Istituto Italiano di TecnologiaTorino, Italy; ^2^Neuroscience and Brain Technologies Department, Istituto Italiano di TecnologiaGenova, Italy

**Keywords:** cybernetics, bio-electronic systems, resistive switching devices, memristors, neuromorphic devices, multielectrode arrays

## Abstract

Here we provide the state-of-the-art of bioelectronic interfacing between biological neuronal systems and artificial components, focusing the attention on the potentiality offered by *intrinsically* neuromorphic synthetic devices based on Resistive Switching (RS). Neuromorphic engineering is outside the scopes of this Perspective. Instead, our focus is on those materials and devices featuring genuine physical effects that could be sought as non-linearity, plasticity, excitation, and extinction which could be directly and more naturally coupled with living biological systems. In view of important applications, such as prosthetics and future life augmentation, a cybernetic parallelism is traced, between biological and artificial systems. We will discuss how such intrinsic features could reduce the complexity of conditioning networks for a more natural direct connection between biological and synthetic worlds. Putting together living systems with RS devices could represent a feasible though innovative perspective for the future of bionics.

## Introduction

The brain is the most powerful and complex known computational system. A recent work evaluates the memory capacity of the human brain to be in the order of 10^15^ Bytes (Bartol et al., 2016). Unlike the hardware and software of a machine, the mind and brain are not distinct entities, feature that resembles the so called *firmware*. How could we represent a neuronal synapse, a complex structure containing hundreds of different proteins with a single line of code? We still do not know the detailed circuitry of any region of the brain well enough to reproduce its structure and, as a consequence, its behavior (Brooks et al., 2012).

The technological roadmap toward integration of synthetic and biological functions was described in the past as cybernetics, in a definition given by N. Wiener (Wiener, 1961) that recalls ancient Greek κυβερνητική τέχνη, the art of the pilot. This definition moves from the hypothesis that there is a substantial analogy between self-regulation mechanisms in living beings and machines, based on information flow and feedback/closed loop.

Putting the focus on silicon microdevices, we should trace a boundary between (Breslin and O'Lenskie, 2001).

1. *extrinsic neuromorphic systems*, based on CMOS circuits that enable processing of information as occurs naturally in biological brains, including silicon based artificial synapses and artificial neurons sorted in neural networks, that are outside the scopes of this review;
2. *intrinsic neuromorphic systems*, artificial synapses or arrays of elements that inherently possess key figures such as plasticity, non-linearity, spiking processing capabilities.

Giant projects / frameworks, such as the DARPA SyNAPSE program (Systems of Neuromorphic Adaptive Plastic Scalable Electronics) and the EU Human Brain Project deal with standard extrinsic systems and therefore are outside the scopes of this Perspective. Their paradigm is reproducing the configurational complexity by emulating a simplified physical model in an extremely high number of elements (at least 10^10^ neurons and 10^14^ synapses).

## Neuronal networks *in vitro*: a simplified model of brain circuits

The neuronal assembly, as defined by Hebb (1949) is “a group of cells that share similar static and dynamic response properties, constituting the simplest instance of a representative process.” A network of sparsely coupled neurons from different brain areas developing *in vitro* is a useful experimental model for a generic assembly, since it has been proven to retain fundamental properties of the original tissue and the same distribution of cell types (Marom and Shahaf, 2002). Dissociated neurons can be cultured *in vitro* for many months (Potter and DeMarse, 2001) as they form a new 2D network functionally connected by synapses. This experimental model can be easily coupled to substrate-integrated Micro-Electrode Arrays (MEAs). Planar MEAs consists of glass or silicon over which a conductor is patterned in order to design specific layout of electrodes where electrogenic cells can grow and develop (Gross et al., 1977; Maher et al., 1999; Berdondini et al., 2009). They were first developed in the late 70's thanks to the advancements in micro-fabrication technologies. The lab of Prof. G. Gross at the University of North Texas has pioneered in the development of microelectrode arrays to be coupled to neuronal networks (Gross et al., 1977; Gramowski et al., 2000). Nowadays these devices allow *in vitro* (Jimbo and Kawana, 1992; Gross et al., 1995; Massobrio et al., 2015) and also *in vivo* (Vassanelli et al., 2012; Vassanelli, 2014) multi-site, long-term recordings of the activity of neuronal populations and extracellular stimulation from one or more electrodes of the array. Further, advancements of this technique allow nowadays recording and stimulating from hundreds/thousands of electrodes (Kaul et al., 2004; Heer et al., 2006; Pearce and Williams, 2007; Frey et al., 2009; Thewes et al., 2016), and simultaneously record electrical and optical signal through transparent diamond electrodes (Ariano et al., 2005, 2009). Multiparametric measurements are suitable for research devices and laboratory investigation while the biological electrical activity and MEA-like devices appear, at a glance, the most suitable technology that should be easily wearable and provide comfort in addition to functionality (Stoppa and Chiolerio, 2014).

Dissociated neurons in culture show spontaneous electrical activity that can be easily measured and evaluated through the use of MEAs. The firing rate of the cells changes during the *in vitro* development, related to the age of the network (Van Pelt et al., 2004; Bologna et al., 2010). Starting from the second week in culture, spikes tend to cluster into bursts, thus presenting a kind of activity which persists for the whole life span and represents a mature state of the network (Maeda et al., 1995; Bonifazi et al., 2005; Chiappalone et al., 2006, 2007; Eytan and Marom, 2006; Biffi et al., 2013; Bisio et al., 2014). The “bursting” mode of activity can be also modulated *in vitro* by appropriate electrical and/or chemical stimulation. In general, low frequency, sustained electrical stimulation locks the phase of periodic bursts to the applied stimuli (Maeda et al., 1995). Higher rates of stimulation induce a transition from synchronized bursting activity into a more sparse spiking behavior (Wagenaar et al., 2005). In particular, *in vitro* experiments in different neural preparations have shown that Hebbian plasticity, in the form of long-term potentiation and depression, provides the basis of many models of learning and memory (Shahaf and Marom, 2001; Chiappalone et al., 2008; Le Feber et al., 2010; Stegenga et al., 2010, and for a complete review see Massobrio et al., 2015).

For the above reasons, neuronal networks represent a very powerful yet simple and easy accessible system that retains important properties of the original brain tissue. Coupled to electronic devices that can read, process and stimulate their electrophysiological activity, they form the so called “bio-artificial living systems.” These innovative hybrid systems are now paving the way for next generation of neural interfaces and intelligent neurally-inspired information processing systems.

## Hybrid interfaces and closed-loop systems: toward bio-electronic computational systems

**Figure 1:**
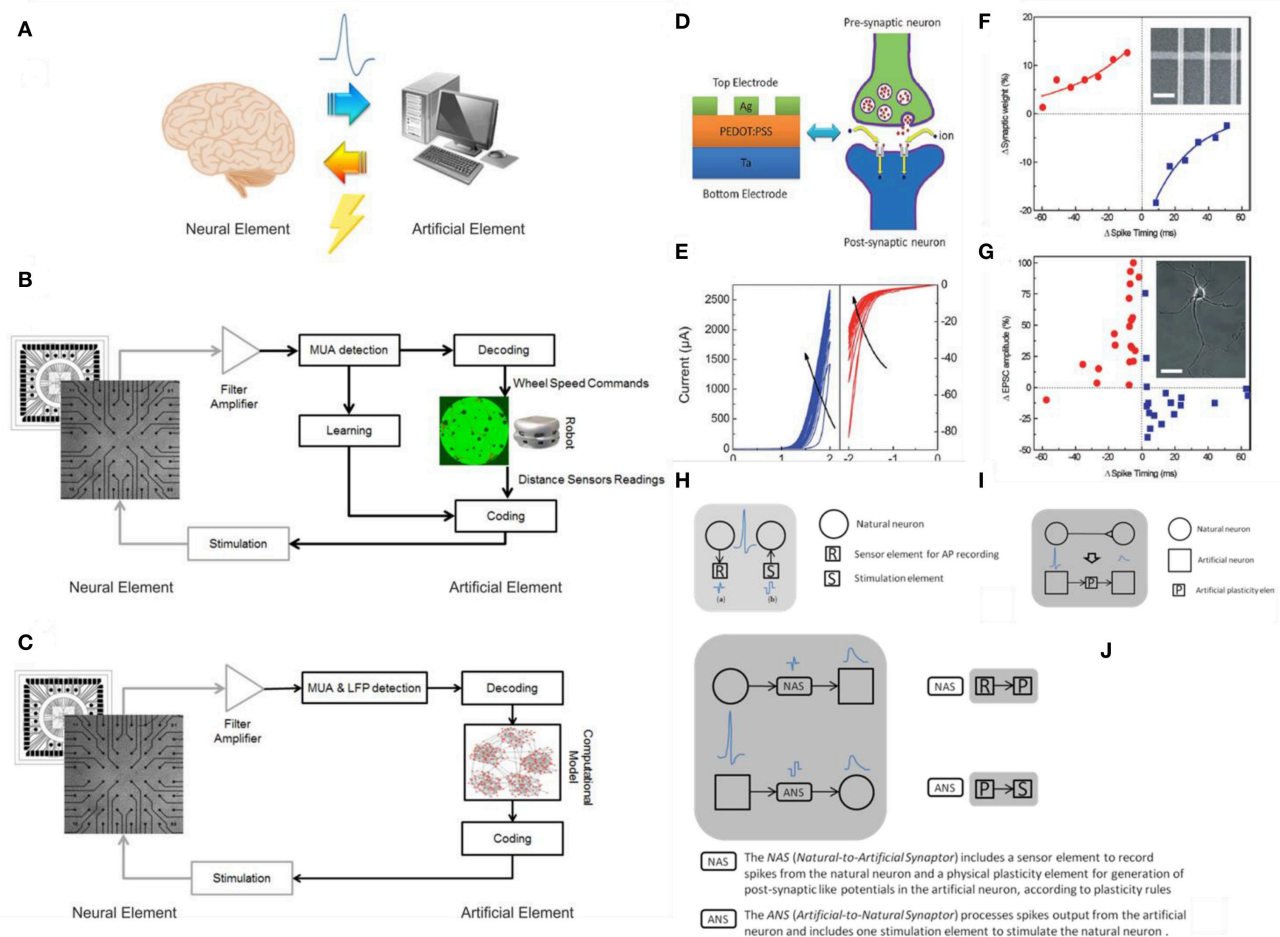
**Hybrid systems. (A)** A cartoon of a neural hybrid system, which is typically composed by a neural element (the brain or a simplified model of it) and an artificial one (a computational device, in general). The two elements communicate through a bi-directional interaction realized through the acquisition of the “biosignals” from the neural element to the artificial one and, after data processing, a specific stimulation pattern is fed back to the neural element. **(B)** An example of neurorobotic system where a culture of dissociated neurons is able to bi-directional interact through a signal processing block (Multi-Unit-Activity, MUA, detection) with either a physical or a virtual robot (modified from Tessadori et al., 2012). **(C)** An example of bidirectional interaction between a biological network coupled to a MEA and a computational model of a neural network: this experimental framework can be exploited for medical applications such as innovative cognitive/brain prostheses (modified from Bonifazi et al., 2013). The communication between the artificial element and its neuronal counterpart is accomplished by the “Coding” and “Decoding” blocks in both panels **(C,D)**. **(D)** Analogy between a multilayered hybrid memristor and a biological synapse: structural analogy in a Ag/PEDOT:PSS/Ta stack and biological synapse. **(E)** IV curves obtained after multiple consecutive scans. Panels **(D,E)** reprinted with permission from Li S. et al. (2013). **(F)** Comparison between synthetic and biological STDP measurements: synaptic weight as a function of the synchronization timing between pre and post-synaptic signals in a network of memristive devices. **(G)** STDP effect in living biological neurons. Panels **(F,G)** reprinted with permission from Jo et al. (2010). **(H)** Communication to real physical neurons established through micro –nanoelectronic components. **(I)** Memristive synapse: a physical plasticity component is developed to emulate natural synapse behavior. **(L)** Synaptors: signal transmission between artificial and natural neurons. Panels **(H–L)** adapted from http://www.rampproject.eu/project-objectives, last accessed May 26th 2016.

A hybrid system is defined as the combination between a biological and an artificial element (Figure 1A). Typically a biological element is able to “talk” to an artificial one thanks to specific algorithms that can translate the language of the cells into commands or instructions (Mussa-Ivaldi et al., 2010). In neuroscience in particular there has been a growing interest for “closed-loop” experiments, in which recordings of various types are used to modulate stimulation (Mavoori et al., 2005; Jackson et al., 2006; Venkatraman et al., 2009). The underlying rationale is that the dynamic and adaptive properties of neural systems can be understood by looking at their interaction, in a bi-directional closed-loop, with their external environment. Moreover, the environment itself can be manipulated, and the changes in dynamic behavior resulting from changes in the environment provide useful information for understanding the neural systems themselves. These experimental paradigms allow manipulations of the neural system under study that once were only possible with simulations on detailed computational models. “Biological” closed-loop experiments (Figures 1B,C) have been performed at single neuron level, by interfacing artificial and actual neurons (Le Masson et al., 2002) at population level, by controlling the dynamic regime of neuronal populations (Wagenaar et al., 2005), and by investigating basic mechanisms of learning (Shahaf and Marom, 2001; Le Feber et al., 2010) and at the level of a “kind of whole organism,” in experiments in which portions of nervous tissue are connected to artificial or virtual (Reger et al., 2000; DeMarse et al., 2001; Kositsky et al., 2009), or artificial/hybrid animals (Novellino et al., 2007; Tessadori et al., 2012). It was found that unidirectional (open-loop) periodic perturbation mode resulted in entrainment loss, while bidirectional (closed-loop) mode is able to maintain the phase-locked entrainment (Jung et al., 2001). Closed-loop experiments are also relevant to the technology of neural interfaces (Mussa-Ivaldi and Miller, 2003; Nicolelis, 2003; Berger et al., 2011; Bonifazi et al., 2013). In fact, the latter implies the ability to monitor neural activity at population level in real-time and, conversely, to generate patterns of time- and space-varying stimuli, again in real-time.

These results prove that a connection between biological and artificial systems is possible creating a conditioning black box, in other words a translator that adapts the time-, space-, amplitude, and shape characteristics of electrical stimuli (Vassanelli and Mahmud, 2016). Clearly the more intrinsically neuromorphic a synthetic network is, the less complex this conditioning bottle-neck will be. Hence Resistive Switching Devices (RSDs), that possess unique properties in this respect, well represent to date the most advanced condensed matter candidate for a direct coupling with living neuronal systems. Nevertheless interfacing living biological brains is still a though target to be achieved, we are experiencing the first recent promising results (Gupta et al., 2016a). Worth mentioning also the strong effort in finding soft solutions for electrodes featuring a complete matching (not yet announced) of mechanical properties with biological tissues (Rogers et al., 2016), or the many attempts of interfacing the peripheral nervous system for the control of neuroprostheses and hybrid bionic systems (Navarro et al., 2005).

## Intrinsic neuromorphic properties of RSDs and coupling with living neurons

The best prototype of an intrinsic neuromorphic system is perhaps the *memristor*, a promising solution for the beyond-Moore era of nanoelectronics. In 1971, L. O. Chua predicted the existence of a fourth fundamental device (meaning that it cannot be realized using passive devices), which he called a “memristor,” contraction of “memory resistor” (Chua, 1971). Since then, many RSDs were developed exploiting the properties of a plethora of nanoscale materials (Calzecchi-Onesti, 1884; Sawa, 2008; Strukov et al., 2008; Pershin and Di Ventra, 2011; Yang et al., 2013, 2016; Lin et al., 2014; Porro et al., 2015; Laurenti et al., 2017), even in liquid form (Chiolerio et al., 2016).

Currently we talk about RSDs in place of memristors, as the diverse mechanisms that enable device switching do not feature the ideal properties required by Chua's ideal bipole (Vonger and Xiangkang, 2015).

RSDs were proposed as neuromorphic emulators (Snider, 2008; Pershin et al., 2009). Crossbar RSDs network can potentially offer connectivity similar to neurons with RSDs working as synapses. Moreover the function density (10^10^/cm^2^) comparable to those of biological systems can be potentially obtained with advanced lithography approaches. Regarding neuromorphic functionalities that can be found in artificial systems, RSDs were shown to possess:

1. short term plasticity (STP);
2. long term plasticity (LTP);
3. spike-timing dependent plasticity (STDP);
4. spike-rate dependent plasticity (SRDP).

STP is the temporal potentiation of synaptic connections, lasting from seconds to minutes and then fading away with the ceasing of the stimulus. LTP is a more prolonged potentiation, lasting for years or even permanent. STDP is thought to be the most important feature of synaptic plasticity in biological brains. It implies that the synaptic weight varies according to the timing of the pre- and post-synaptic spikes. If a post-synapse is stimulated after a pre-synapse (Δ*t* < 0), the synaptic weight is increased and produces a long-term effect (LTP). If the order is reversed (Δ*t* > 0), the synaptic weight drops and there is a long term depression (LTD). Furthermore, the strength of synaptic plasticity is proportional to the pre-synaptic spiking rate (SRDP). (Jo et al., 2010; Li S. et al., 2013; Chen et al., 2014).

The first confirmation of STDP was obtained from Jo et al. (2010) using a metal/insulator/metal (MIM) RSD that works by migration of silver ions with the formation of conductive metallic filaments. Also Li S. et al. (2013) invoked the formation of metallic filaments in this case the matrix was a conductive polymer PEDOT-PSS. While the LTP is believed to be due to the growth of the silver filaments, the PEDOT-PSS is responsible for the STP, STDP, and SRDP (Figures 1D–G). The formation of metallic filaments is believed to be hindered by the elastic recovery of the polymer while a “high-rate stimulation with either a high strength or high frequency will enhance ion movement, suppress the elastic recovery and then result in the LTP.”

The retention of RSDs based on metal oxides decays exponentially, Chang et al. compare the memory decay of tungsten oxide RSDs to human memory loss (Chang et al., 2011). Wang et al. worked on amorphous indium gadolinium zinc oxides (Wang et al., 2012) demonstrating essential synaptic functions including SRDP, STDP, LTP/STP, and “learning-experience” behavior. The STP decay is explained with the relaxation processes determined by the back-diffusion of oxygen ions. The LTP is enhanced through high-rate stimulation with short time interval and repetitive stimulation training demonstrating thus SRDP and STDP. Subramaniam et al. integrated two different devices in the same circuit: TFT nanoparticles were used to produce STP and SRDP by reacting to pre-synaptic spike signals while metal oxide RSDs based on hafnium oxide was used as memristive device to simulate LTP (Subramaniam et al., 2013). This kind of system is now very popular and synapses analogs were fabricated using different types of metal oxides RSDs as reported in Table 1.

**Table 1:** **Resistive Switching Devices intrinsic neuromorphic properties in a comparison table, according to existing literature**.

**Family**	**STP**	**LTP**	**STDP**	**SRDP**	**Type**	**References**
Metal filament			X		Array	Jo et al., 2010
	X	X	X	X	Single	Li S. et al., 2013
Metal oxide	X	X			Single	Chang et al., 2011
	X	X	X	X	Single	Wang et al., 2012
		X	X		Single	Williamson et al., 2013
	X	X	X		Array	Subramaniam et al., 2013
			X	X	Single	He et al., 2014
		X	X		Single	Kim et al., 2015
			X		Array	Wang Y.-F. et al., 2015
			X		Single	Du et al., 2015
			X		Array	Wang Z. et al., 2015
			X		Array	Matveyev et al., 2016
			X		Array	Prezioso et al., 2016
Spintronic		X	X		Single	Krzysteczko et al., 2012
			X		Single	Wang et al., 2014
			X		Array	Kaneko et al., 2014
Chalcogenide	X	X	X		Array	Ohno et al., 2011
	X	X	X		Single	Nayak et al., 2012
			X		Array	Kuzum et al., 2012
			X		Single	Li Y. et al., 2013
			X	X	Single	Li et al., 2014

Krzysteczko et al. demonstrate that MTJ (Magnetic Tunnelling Junction) RSDs intrinsically exhibit the features of biological synapses and neurons (Pershin and Di Ventra, 2008). Repeated treatment with voltage pulses led to a gradual, non-volatile change in MTJ resistance emulating LTP and LTD. Voltage-induced resistance variation is described by flux providing the scope for the emulation of STDP. Similar results were obtained from Wang et al. (2014) while this kind of RSD was used to build an on-chip pattern recognition of a multishaded grayscale image in a neural network circuit with multiple neurons (Kaneko et al., 2014).

The charge transferred under pulsed operation to an RSD compared with the amount exchanged by synapses is usually quite high. Also the electric potential of the spike is typically at least one order of magnitude higher. Yet, recent findings demonstrate that soft materials are able to set and reset in a narrow voltage range comprised between 50 and 100 mV (Rajan et al., 2016) with On currents as low as 40 μA and Off currents of 600 nA (Rajan et al., 2017), that could easily be matched to induce living neurons through an impedance load connected in parallel. The main problem in coupling RSDs networks with living neurons is the higher range of power used to change RSD state, in the range of 250 μJ (Gupta et al., 2016b) and the low energy and power density of neurons. Indeed up to now there are two reports showing the first experimental realization of an hybrid network of living neuronal cells connected to a layer of artificial spiking neurons (Gater et al., 2014; Gupta et al., 2016a). Quite similar systems were shown to result in a multistate resistive switching, hence reducing the distance between neuromorphic devices and biological brains (Sandouk et al., 2016). Closed-loop systems may be realized by modulating the compliance current given as input to the RSDs network using as template the action potential of the biological synapse network, realizing a point-to-point spatial equivalence between the two networks and obtaining a dynamically coupled pair of non-linear oscillator networks (Jung et al., 2001).

We conclude that it is natural to expect a wide integration between the biological and the synthetic worlds (Demin et al., 2015; Wu et al., 2015; Feali and Ahmadi, 2016). Funding and research efforts are currently experiencing a slow shift from the focus on neuromorphic engineering and an extrinsic paradigm of emulation of biological processes (to cite some, project CORONET http://cordis.europa.eu/project/rcn/97109_en.html) belonging to FP7, EU), to more open frameworks where the use of RSDs could help in closing the loop (such as RAMP project, http://www.rampproject.eu, EU, Figures 1H–L). Referring to this, we read “*Artificial neural networks in the form of software run on conventional von Neumann computers appear incomparable to the biological systems* in terms of speed, energy efficiency, adaptability, and robustness. The challenge is to build a physical neural network where elements overcome this deficiency by merging data storage and processing into single electronic devices […] we aim to create a new biohybrid architecture of tightly coupled natural and artificial neurons endowed with plasticity properties. […] *Adaptation properties of the artificial network will rely on memristive nanoelectronic devices with synaptic-like plasticity* and on activity-dependent rearrangement of neuronal connectivity. As such, the biohybrid system will provide new and unique adaptive, self-organizing and evolving properties deriving from the fusion of natural and artificial neuronal elements into *a new plastic entity*” The RAMP project reports the specific target that this Perspective is addressing: the “Development of physical components with plastic properties based on nano-scale memristors in combination with CMOS circuitry emulating the function of a biological synapse.” Nevertheless we see, once more, that CMOS emulators are required to achieve the so called Synaptor, a new biohybrid signal transmission unit that couples together one natural and one artificial neuron.

We may also imagine that the direct coupling between two perfectly compliant neuromorphic systems could lead to spontaneous adaptation, locking and growth of the networks, that will behave as a single super-network and exchange freely information.

## Conclusions

After being theorized 40 years ago, it is only in the last decade that the fabrication of intrinsically neuromorphic devices was demonstrated. Following this milestone, in the latest years relevant efforts in the scientific community was directed toward the development of new materials for RSDs as well as theoretical algorithms for their use. One of the most promising applications of RSDs is in developing neuronal networks.

On the other hand in the recent years it was possible to grow neurons over artificial substrates and new methodologies for the activity recording allowed the study of signals in neuronal networks and direct interaction with bio-artificial circuits, with a specific care in the simultaneous recording of signals from a complex network of neurons, in place of a single and isolated cell.

Coupling RSDs with neuronal networks is still a distant objective. Nowadays we are at the edge of a new era where it will be possible to conceive and develop systems with reliable electrical interfaces between the brain and RSD-based neuronal networks, with the possibility of integrating them in wearable and comfortable devices. Neural prosthetics will be interfaced directly without any computer translation and used to fight serious neurological conditions resulting from disease, aging, or injury. And what about enhancing cognitive capacities of healthy people? The logical next step.

## Author contributions

All authors gave substantial contribution to the development of this work equally, drafting and revising it critically; furthermore all Authors approved its final version for publication.

## Funding

This work was fully funded by Fondazione Istituto Italiano di Tecnologia.

### Conflict of interest statement

The authors declare that the research was conducted in the absence of any commercial or financial relationships that could be construed as a potential conflict of interest.
